# Editorial: Wellbeing and adherence to physical activity: What are the factors of the wellbeing concept leading to exercise adherence?

**DOI:** 10.3389/fspor.2022.1035259

**Published:** 2022-10-04

**Authors:** Hans-Peter Kubis, Matteo Bonato, Francesco Sartor

**Affiliations:** ^1^School of Human and Behavioural Sciences, Bangor University, Bangor, United Kingdom; ^2^Department of Biomedical Sciences for Health, Università degli Studi di Milano, Milan, Italy; ^3^IRCCS Istituto Ortopedico Galeazzi, Milan, Italy; ^4^Department of Patient Care & Monitoring, Philips Research, Eindhoven, Netherlands

**Keywords:** physical activity, adherence, wellbeing, mental health, quality of life

The Frontiers Research Topic entitled: “*Wellbeing and adherence to physical activity: what are the factors of the wellbeing concept leading to exercise adherence?*” is aimed at advancing the evidence around the association between wellbeing factors and physical activity adherence.

Physical activity (PA) delays all-cause mortality in the general population, reducing the risk of developing chronic diseases (1). Nevertheless, long-term adherence to PA and exercise programs, which is the key to such earned health and psychological benefits, is still low, with large proportions of the population, with higher numbers in certain groups, failing to meet the recommended amount of PA (2). The association between physical activity and wellbeing, however, does not explain why some people adhere to exercise and some do not. Despite the well-documented issues with long-term exercise adherence and exercise dropouts, few data exist regarding the factors associated with maintaining exercise behavior.

Regarding exercise adherence, the study of Bazett-Jones et al. investigated whether the social distancing restrictions during the 2020 COVID-19 pandemic affected exercise motives, socialization, wellness, and mental health in youth long-distance runners (9–19 years old). Through a customized online questionnaire, demographics, motive for running, and wellness (sleep quality, anxiety, running enjoyment, food consumption quality) 6 months before, as well as during, social distancing restrictions during the COVID-19 pandemic were investigated. Authors found that youth long-distance runners were negatively impacted by COVID-19 social distancing restrictions, which potentially had implications for health and wellbeing. Youth long-distance runners experienced changes in motivation, lower enjoyment of running, changes in wellness, and more anxiety even though they were not necessarily restricted in their ability to run, only the context and reasons for participation. Moreover, running distance and frequency were reduced, which may be affected by the removal of competition and social motivators.

The type of exercise plays an important role in affective responses and participation in sports. A potential exercise type for improvement of sport participation and affective and enjoyment responses could be the use of Sprint Interval Training (SIT) (Hu et al.). SIT is a training method characterized by intensity of “all-out” effort and superior time-efficiency compared to traditional moderate-intensity continuous training (MICT). In their systematic review and meta-analysis, Hu et al. investigated all related SIT studies in the literature and comprehensively evaluated the affective and enjoyment responses to SIT in healthy populations in comparison to MICT and other high-intensity interval training protocols (HIIT). Analyzing the 25 studies included in the review, authors found that there is a wide range of demonstrated affective and enjoyment responses of SIT with differences in exercise configuration in healthy individuals. Overall, the results in this review demonstrated that SIT elicits negative and lower affective valences compared to MICT immediately post-exercise, but similar low affective valences during exercise and a comparable post-exercise affective state to MICT or HIIT. Moreover, enjoyment responses in SIT were comparable to MICT or HIIT in healthy individuals, suggesting similar future adherence in SIT with better time efficiency than MICT or HIIT. Based on the available literature and data at this time, authors suggested that it is still premature to conclude on whether SIT, or which SIT configuration, could induce more positive affective responses. Likewise, authors emphasize that there is currently inadequate data to be able to conclude which exercise modality is going to be more likely to be adhered to due to the limited age groups included and inconsistent research design across studies involving SIT protocols, as well as individual differences among the participants. In conclusion, this review by Hu et al. suggests that it might be important to consider adopting low volume SIT protocols with shorter sprint duration and fewer sprint repetitions and incorporating music and group exercise to reduce adverse affective responses, especially when introducing SIT to individuals with low tolerance to high-intensity exercise. Moreover, given that physiological improvements elicited by SIT are not attenuated with shorter and fewer sprints, low-volume SIT may produce more positive affective responses and possibly higher future adherence, being a better choice to maximize the time-efficiency of SIT.

Although the close positive relationship between wellbeing and exercise adherence has been confirmed by numerous studies, it is still unclear whether this relationship exists for children and adolescents, because previous research mainly focused on adults. To this regard, the systematic review, provided by Chen and Wu aimed to explore what the wellbeing factors are that might contribute to exercise adherence and how these factors shape exercise adherence for children and adolescents. After systematically filtering and categorizing the extant studies adopting an either quantitative or qualitative approach, only seven studies were considered. Based on these studies, the results showed that the positive relationship between wellbeing and adherence was not as stable and reliable to impact on exercise adherence. In fact, the number of participants, sensitivity and reliability of exercise adherence measurement, gender, and health conditions for children and adolescents might influence the relationship between wellbeing and exercise adherence. Particularly, parental support and supervision should also be conducted with caution and adjusted to the differential needs of various children and adolescents.

Finally, the study by Gong and Sheng, investigated the differences in parameters of exercise health beliefs among college students of different genders, and assessed the relationship between demographic factors and parameters of exercise health beliefs i.e., to examine the relationship between exercise self-efficacy and internal components of exercise health beliefs. Results showed that, compared with female students, male students have higher perceived benefits and self-efficacy of exercise and lower perceived subjective and objective barriers. Monthly family income had a significant positive correlation with exercise self-efficacy and a negative correlation with perceived subjective barriers to exercise disorder. Exercise self-efficacy was positively correlated with perceived benefits and perceived severity and a significant negative correlation with perceived subjective and objective barriers was observed. Within the exercise health belief items, there was a negative relationship between perceived subjective barriers and exercise self-efficacy. In conclusion, findings provided a new psychological angle for understanding the exercise condition of college students and the restraining factors and provided new insights into increasing exercise self-efficacy to lower the subjective barriers to exercise.

In conclusion, the evidence provided by this Research Topic indicates that wellbeing benefits expectation can drive, by itself, PA adherence in a highly intrinsically motivated population, such as the youth long-distance runners investigated in Bazett-Jones et al. This is true even when, and this is the striking fact, the competitive and socialization elements are removed. However, when a young population not as equally intrinsically motivated was considered, wellbeing did not seem to drive PA adherence (Chen and Wu). If it was not surprising that intrinsic motivation plays such a decisive role in engaging in PA, it is, nevertheless, still key to investigate the main perceived barriers that hinder the link between wellbeing and PA adherence, as well as the strategies to lower such perceived barriers (Gong and Sheng). Future research should try to answer the following questions: How can we, as exercise physiologists and coaches, make PA more enjoyable to those who do not have a high intrinsic motivation to start with? Or, as shown by Hu et al., what is the exercise routine that can maximize the cost-benefit ratio?

## Author contributions

MB wrote the first draft of the present manuscript. H-PK and FS contributed to its critical revision. All authors contributed to the article and approved the submitted version.

## Conflict of interest

Author FS works for Philips Electronics Nederland B.V., which provided support in form of salary, but did not have any additional role in the content, decision to publish, or preparation of this work. The remaining authors declare that the research was conducted in the absence of any commercial or financial relationships that could be construed as a potential conflict of interest.

## Publisher's note

All claims expressed in this article are solely those of the authors and do not necessarily represent those of their affiliated organizations, or those of the publisher, the editors and the reviewers. Any product that may be evaluated in this article, or claim that may be made by its manufacturer, is not guaranteed or endorsed by the publisher.
